# Financing surgical, obstetric, anaesthesia, and trauma care in the Asia–Pacific region: proceedings

**DOI:** 10.1186/s12919-023-00256-z

**Published:** 2023-07-25

**Authors:** Rennie X. Qin, Sangchul Yoon, Zachary G. Fowler, Anusha Jayaram, Makela Stankey, Lubna Samad, Kiki Maoate, Kee B. Park

**Affiliations:** 1 The Program in Global Surgery and Social Change, the Department of Global Health and Social Medicine, Harvard Medical School, 641 Huntington Ave, Boston, MA 02115 USA; 2 Department of Medical Humanities and Social Sciences, College of Medicine, Yonsei University, Seoul, South Korea; 3 Tufts University School of Medicine, 145 Harrison Ave, Boston, MA 02111 USA; 4 Keck School of Medicine at the University of Southern California, 1975 Zonal Ave, Los Angeles, CA 90033 USA; 5 Interactive Research and Development (IRD), 4Th Floor, Woodcraft Building, Plot 3 & 3 A Sector 47, Korangi Creek Road, Karachi, Pakistan; 6 Department of Surgery, University of Otago, 2 Riccarton Avenue, Christchurch Central City, Christchurch, 8011 New Zealand

**Keywords:** Global surgery, Surgical system strengthening, National surgical planning, Partnership, Asia–Pacific

## Abstract

Surgical, obstetric, and anaesthesia care saves lives, prevents disability, promotes economic prosperity, and is a fundamental human right. Session two of the three-part virtual meeting series on *Strategic Planning to Improve Surgical, Obstetric, Anaesthesia, and Trauma Care in the Asia–Pacific Region* discussed financing strategies for surgical care. During this session, participants made a robust case for investing in surgical care given its cost-effectiveness, macroeconomic benefits, and contribution to health security and pandemic preparedness. Funding for surgical system strengthening could arise from both domestic and international sources. Numerous strategies are available for mobilising funding for surgical care, including conducive macroeconomic growth, reprioritisation of health within government budgets, sector-specific domestic revenue, international financing, improving the effectiveness and efficiency of health budgets, and innovative financing. A wide range of funders recognised the importance of investing in surgical care and shared their currently funded projects in surgical, obstetric, anaesthesia, and trauma care as well as their funding priorities. Advocacy efforts to mobilise funding for surgical care to align with the existing funder priorities, such as primary health care, maternal and child health, health security, and the COVID-19 pandemic. Although the COVID-19 pandemic has constricted the fiscal space for surgical care, it has also brought unprecedented attention to health. Short-term investment in critical care, medical oxygen, and infection prevention and control as a part of the COVID-19 response must be leveraged to generate sustained strengthening of surgical systems beyond the pandemic.

## Introduction 

In the second session of the three-part meeting series on *Strategic Planning to Improve Surgical, Obstetric, Anaesthesia, and Trauma Care in the Asia–Pacific Region*, 215 participants from 42 countries convened to discuss funding strategies for surgical system strengthening. The session opened with framing statements on the cost-effectiveness and macroeconomic benefits of investing in surgical care, strategies for creating fiscal space, and the impact of the COVID-19 pandemic on fiscal space for surgical care. This was followed by presentations by a range of funders, including development banks, bilateral donors, philanthropic organisations, and innovative financing. Table 1 summarises the funders’ priorities, projects, and areas of work. This session was moderated by *Lubna Samad, Director of Global Surgery Programs at IRD, Pakistan, and Kiki Maoate, paediatric surgeon at Christchurch Hospital and President of the Pasifika Medical Association, New Zealand.*

**Table 1:** Funders’ key messages, priorities, projects, and countries of interest

Type of funder	Name of funder	Countries/region	Key messages, priorities, and projects
Develop-ment banks	Islamic Development Bank Group (IsDB)	Central & Southeast Asia	Primary health care Expanding UHC to vulnerable populations Quality & equitable community healthcare Establishing social health insurance One health
Financing institutions	Global Financing Facility for Women, Children, and Adolescents (GFF)	Not stated	Health outcomes for women, children, and adolescents Health system resilience Developing long-term national health plans
	International Finance Corporation (IFC)	Not stated	Private sector engagement in healthcare
Bilateral agencies	Korea International Cooperation Agency (KOICA)	14 countries in the Asia–Pacific region	Increasing support for health projects under the New Southern Policy Delivering high-quality maternal, child, and newborn care services through equipment provision, workforce training, and blood system strengthening
	Japan International Cooperation Agency (JICA)	The Asia–Pacific region	Leaving no one behind Health security Infectious disease surveillance Building hospitals through ‘hard’ and ‘soft’ assistance
	United States Agency for International Development (USAID)	Not stated	Infectious disease, maternal and child health, and nutrition MOMENTUM program, including Caesarean sections and childbirth-associated hysterectomies. Surgery for trachoma and lymphatic filariasis hydrocele
Philanthropic organisation	Nick Simons Institute	Nepal	Workforce strengthening in rural areas, scholarships, training, task-shifting
Innovative financing	Global impact partners	Not stated	Impact investment advisory, outcome-based financing

## The case for investing in surgical care

*Jim Yong Kim, Vice Chairman and Partner of the Global Infrastructure Partners and 12*^*th*^* President of the World Bank Group (WBG),* witnessed the importance of building robust health systems that are resilient to external shocks and threats throughout his career, especially in responding to the 2014 Ebola outbreak during his tenure as the WBG President. Today, countries with robust health systems, science-based leadership, and experience with previous pandemics have suffered far less economic impact from the COVID-19 pandemic.

In 2019, The WBG initiated the Human Capital Project and discovered that investments in health and education were among the strongest drivers of economic growth over the past 30 years [1]. Investment in surgical care saves lives, prevents disability, and boosts prosperity. Surgical care at the district level increases economic activity by as much as tenfold. The cost of increasing surgical capacity is considerable, yet it pales in comparison to the cost of productivity loss in the absence of adequate surgical care. An estimated investment of $350 billion is required worldwide to increase surgical capacity by 2030; however, an estimated $12.4 trillion will be lost in the gross domestic product (GDP) in low- and middle-income countries (LMICs) if capacity is not expanded [2].

Describing the current moment as a crossroads, Kim urged governments, funders, intergovernmental organisations, and civil society representatives to work together to mobilise investment for surgical system strengthening from both domestic and international sources. Beyond feasibility and cost-effectiveness, surgical care is fundamental to ensuring basic human rights and shared prosperity.

Born in the Republic of Korea, Kim stated his pride in the effective COVID-19 response and the spirit of solidarity demonstrated by many Asia–Pacific countries.

## Macroeconomics of global surgery

*Blake Alkire, Head and Neck Surgeon, Massachusetts Eye and Ear, and Assistant Professor, Harvard Medical School*, discussed the macroeconomic impact of surgery based on economic impact assessment (EIA).

### Economic impact assessment

EIAs focus on the consequences of disease and injury on economic well-being [3–6]. They explore macroeconomic issues of potential interest to stakeholders, policymakers, and donors, such as:

• the macroeconomic cost of diseases to society, at both country and global levels,
• the impact of diseases on a country’s future economic growth,
• the potential economic return on investment of reducing premature deaths by investing in a national strategy to eradicate a disease.

They are different from cost-effectiveness and cost-utility analyses and cannot be used to inform resource allocation in isolation [7].

### Economic welfare

EIAs are rooted in economic welfare, which is interested in an individual or group’s standard of living or general prosperity. Economic welfare depends on people’s ability to consume goods and services, use leisure time as they like, and their overall health status.

Diseases and injury can affect economic welfare by directly reducing health status, impairing the ability to use leisure time in preferred ways, and indirectly affecting the ability to save and invest through reduced labour productivity and out-of-pocket expenditures [4].

### Value of statistical life

In the absence of formal market-based values, economists rely on ‘value of statistical life’ (VSL) to assign monetary values to leisure time and health status [8]. VSL determines how much an individual or a society is willing to pay to reduce mortality risk. If an individual is willing to pay $6 to reduce their chance of dying this year by one in one million, then their VSL is $6/1,000,000 or $6 million. Neither does a VSL of $6 million signify that an individual’s life is worth $6 million, as life has infinite value, nor does it indicate that an individual would pay $6 million to avert their death or accept $6 million in exchange for their mortality. Instead, this VSL implies that 1 million similar individuals would collectively pay $6 million to eliminate the risk that one of them will die within a year randomly.

### Application of macroeconomics approaches

The Lancet Commission on Global Surgery (LCoGS) macroeconomic modelling group aimed to estimate the global macroeconomic burden of common surgical diseases using two economic approaches [2].

1. ‘Value of lost output’ (VLO) examines the disease’s impact on the global market economy in terms of forgone potential GDP. Using the Projecting Economic Cost of Ill-Health (EPIC) model developed by the World Health Organization (WHO), the group projected the market economy from 2015 to 2030 [9]. The EPIC model is based on the Cobb–Douglas function, which relates a country’s economic output with its labour supply, technological progress, and capital stock, such as infrastructure, roads, and buildings.
2. Value of lost welfare’ (VLW) estimates the global economic welfare losses due to surgical diseases in one year. This includes both market losses, such as future income and consumption, and non-market losses, such as the value of health and leisure time. VSL is the basis for determining non-market losses and for conceptualising the impact of factors, including income, health, age, geography, and culture, on an individual or country [10].

**Fig. 1:**
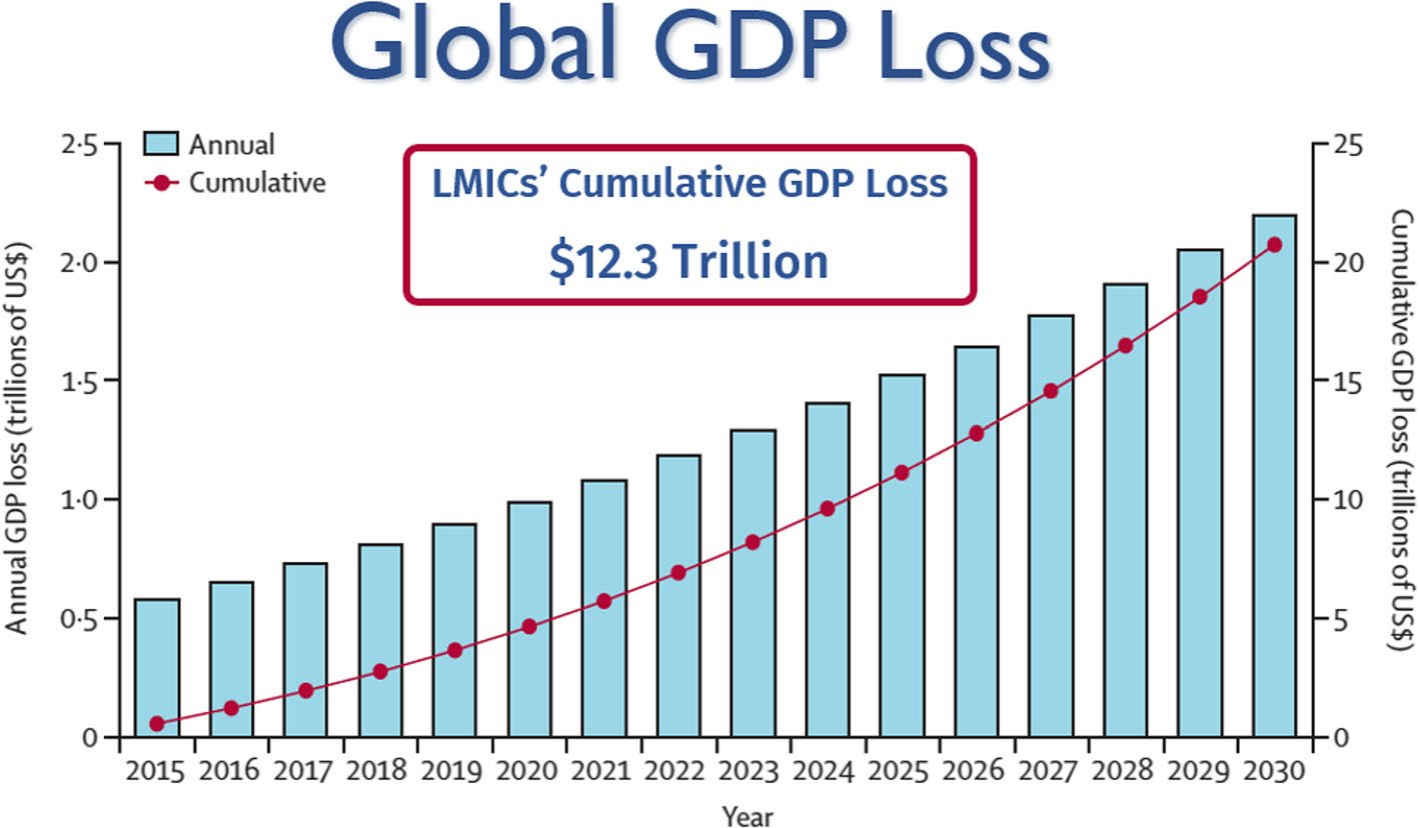
Value of lost output using projecting economic cost of ill-health (EPIC) model: global GDP loss. GDP: gross domestic product; LMICs: low- and middle-income countries

**Fig. 2:**
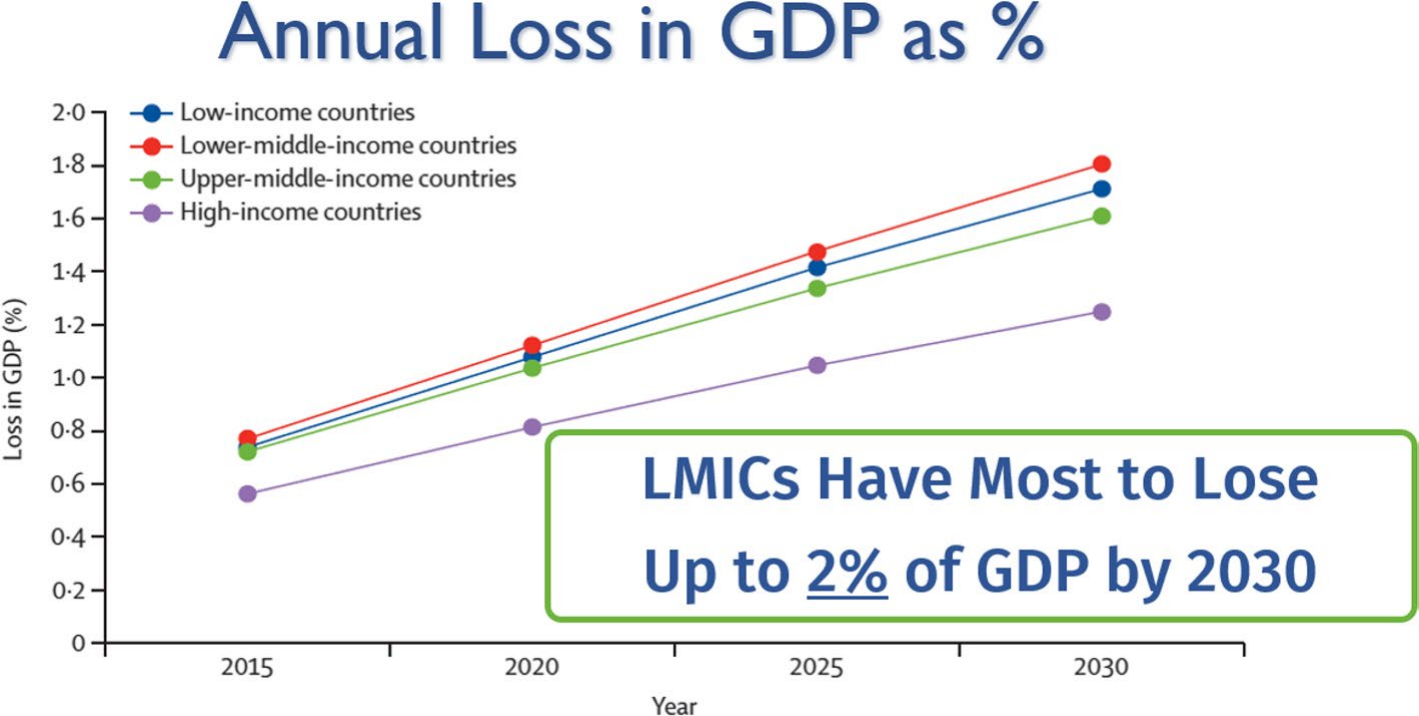
Value of lost output using EPIC model: annual loss in GDP as a percentage. EPIC: projecting economic cost of ill-health; GDP: gross domestic product; LMICs: low- and middle-income countries

The LCoGS used both approaches to perform modelling on five disease categories representing the vast majority of surgical mortality: neoplasm, injury, maternal, neonatal, and digestive disorders. 128 countries had available data for assessing VLO or market economy losses. LMICs risk losing up to $12.3 trillion in GDP from 2015 to 2030 (Fig. 1) and up to 2% of their potential GDP by 2030 (Fig. 2). This figure is higher than similar estimates generated for malaria by an oft-cited study by Jeff Sachs and the WHO Commission on Macroeconomics and Health [11]. Southeast Asia and South Asia risk losing 1.5% of potential GDP by 2030; in Oceania, this risk increases to 2.5%. 175 countries had available data for assessing VLW or economic welfare loss. High-income countries (HICs) had the highest risk of losing the largest equivalent percentage of GDP when all disease categories were combined. However, when neoplasms were removed, LMICs once again faced the greatest risk of impact. Due to the epidemiological transition, LMICs are expected to carry a more substantial portion of the neoplasm burden in the future. Ultimately, the group found that LMICs stood to lose $4 trillion due to surgical mortality in one year alone.

## Methods for creating fiscal space for healthcare

**Fig. 3:**
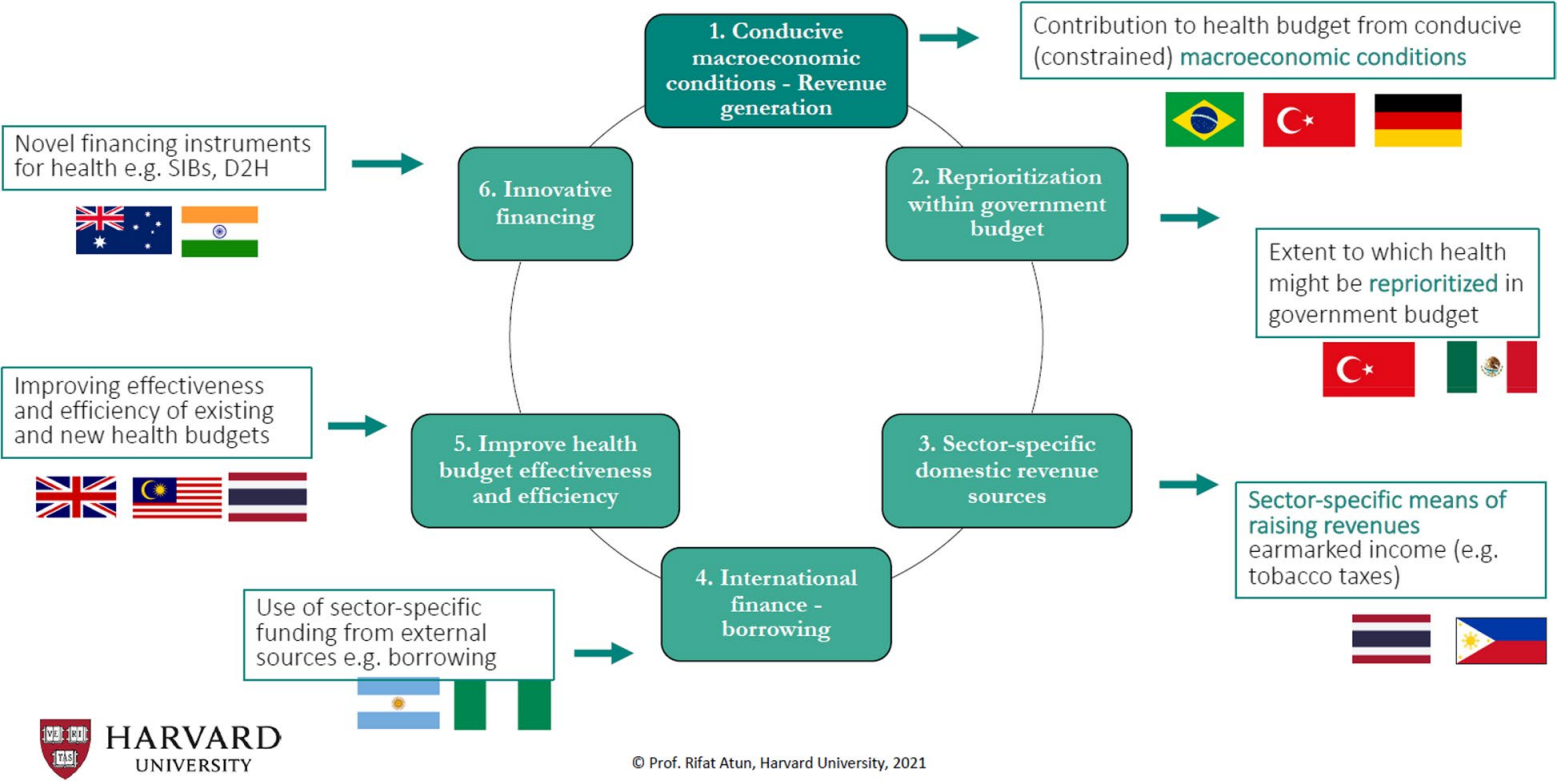
Creating fiscal space for health

*Rifat Atun, Professor of Global Health Systems, Harvard School of Public Health*, noted a critical need to create fiscal space for health. Creating fiscal space is a Sustainable Development Goal (SDG) target for the goal of ending poverty [12]. Its indicator is the ‘proportion of total government spending on essential services (education, health and social protection)’. In fiscal space analysis, six areas of potential sources of financing emerge (Fig. 3) [13].

1. *Conducive macroeconomic conditions*, in which economic growth generates revenue that could be invested in health. Brazil, Turkey, and Germany have utilised this approach.
2. *Reprioritisation of health within government budgets* to allocate more funding to the public health sector. This can be challenging due to the competing priorities of other ministries. However, Turkey and Mexico have successfully reprioritised health in budgeting to excellent outcomes.
3. *Sector-specific domestic revenue*, such as targeted taxes for health. Tobacco taxes have been used in the Philippines to finance universal health coverage (UHC) expansion and in Thailand to fund prevention efforts.
4. *International financing*, in which countries borrow sector-specific funding from external sources. During the COVID-19 pandemic, some countries have been able to borrow at concessional rates to invest in health, including Argentina and Nigeria.
5. *Improving the effectiveness and efficiency of health budgets*. This involves improving the functionality of the health system to achieve greater equity, efficiency, and responsiveness. The United Kingdom (UK), Malaysia, and Thailand introduced priority-setting mechanisms to improve health system functionality. Malaysia’s health system reforms targeted NCDs; Thailand implemented a health technology assessment.
6. *Innovative funding*, such as social impact bonds. Australia and India are utilising innovative funding to expand surgical care.

## Impact of COVID-19 pandemic on health budgets 

*Peter Cowley, Coordinator of the Health Policy and Service Design unit at the World Health Organization Western Pacific Regional Office (WHO WPRO)*, discussed the economic impact of the COVID-19 pandemic. During the pandemic, GDP growth rates decreased by 6.6% in the Pacific, 5.4% in South Asia, and 3.9% in the developing areas of East Asia [14]. Governments are funded by economic activities, such as value-added tax receipts, income tax, and social health insurance contributions [15]. While the actual government revenue declines do not precisely match GDP growth declines, they do resemble them. The revenue shock will be deeper and longer-lasting than in previous crises, such as the 1997 Asian financial crisis and the 2008 subprime mortgage crisis.

Increased expenditures related to COVID-19 further complicate the fiscal space. In most countries, COVID-19 expenses have decreased or stagnated per-capita expenditure on essential healthcare outside of COVID-19, including essential surgical services. Countries that have been able to increase essential healthcare expenditures during the COVID-19 pandemic have more modest increases than pre-pandemic trend lines. In the face of decreasing government revenue, a larger percentage of the budget needs to be allocated toward health to maintain funding levels. COVID-19 vaccination campaigns require additional funding.

Although COVID-19 has shrunken the fiscal space, the substantial investments in increasing intensive care unit (ICU) capacity and ventilator availability will benefit essential surgical services. Across Asia, large numbers of ventilators were purchased through multilateral development bank systems. This movement of capital presents an opportunity for improving surgical care.

## Funder perspectives 

### Development banks 

#### Objectives of the Islamic Development Bank Group (IsDB)

*Ammar Abdo Ahmed, Lead Global Health Specialist, IsDB,* described his bank’s work in Central and Southeast Asia. Most of IsDB’s investment over the past 40 years was channelled toward specialised tertiary-level hospitals. Currently, IsDB is working to redirect support to primary care for IsDB low-income countries and lower middle-income countries. Ahmed emphasised that this does not mean ending IsDB funding for surgical care, as surgical activities related to disability and primary care will be supported. IsDB has selected some of the WHO NCD ‘Best Buy’ interventions to focus on [9]. IsDB is also supporting surgical care through Public Private Partnerships based hospitals.

In shifting the direction of support, IsDB has six new strategic objectives. We could highlight three of them as follow as:

1. Leaving no one behind by expanding UHC package access to vulnerable and impoverished subpopulations in both LMICs and HICs.
2. Providing access to quality and equitable community healthcare services. Surgery-related activities could reduce mortality and morbidity at the community level.
3. Support national health financing initiatives to achieve Universal Health Coverage (UHC)- The Bank will support government programs to achieve the globally recognized objective of financial risk protection through establishing social health insurance to address gaps in UHC coverage.

This effort is part of IsDB’s Lives and Livelihoods Fund, which involves increasing innovative financing and provides concessional financing to IsDB member countries impacted by International Monetary Fund conditionalities. It offers project financing of up to 35% in grant funds and 65% in concessional loans. Projects have included polio eradication efforts in Pakistan and the support of a large, specialised hospital in Tajikistan and Uzbekistan. Discussions are underway regarding projects in Indonesia, Kyrgyzstan, Turkmenistan, Uzbekistan, and all Gulf Cooperation Council countries.

Additionally, One Health is a strategic direction for IsDB during the COVID-19 pandemic. A core mainstreaming of health into all the Bank’s core financing, sectoral and thematic policies is important to transform the weak situation of the health systems and the health outcomes in Member Countries.

### Financing institutions

#### Global financing facility for women, children, and adolescents at the WBG

*Muhammad Ali Pate, Global Director of Health, Nutrition and Population Global Practice at the WBG and Director of the Global Financing Facility for Women, Children, and Adolescents (GFF*), called for leveraging investments in pandemic response toward improving surgical care, for example, through strengthening medical oxygen and improving infection prevention and control. The GFF works with global partners to build resilient health systems and improve outcomes for women, children, and adolescents. These efforts support country leadership in developing national strategic plans and engage with multiple stakeholders who may bring financial support, technical resources, or innovative solutions for safe surgical services delivery.

#### International Finance Corporation (IFC)

*Peggy Tse, Upstream Officer, IFC of WBG*, stated that the IFC focuses on health system affordability, accessibility, and safety, all of which affect the quality of a country’s human capital. She stated that the private sector plays an essential role in this. IFC has developed a broad suite of financing and advisory tools to support the private sector in working more closely with the government to deliver quality health services. Assessing bankability and improving commercial aspects of healthcare projects, IFC works to translate the social impact of these projects into economic benefits, which in turn can mobilise investors. IPC seeks to improve health system resilience through establishing strong links between patient populations and public and private healthcare reimbursement systems. In the absence of a perfect health system, IFC supports committed private sector companies in trying new approaches, targeting ambitious, first-of-a-kind healthcare projects to improve their bankability and commercial viability. Lessons learned are shared across the region, and successful models are replicated and developed as demonstration cases in emerging markets.

### Bilateral agencies

#### Korea International Cooperation Agency (KOICA)

*Do Hyeong Kim, Health Advisor, KOICA,* noted that since 1991, KOICA has implemented projects in 44 country offices, including 14 in the Asia–Pacific region. In 2017, South Korean President Moon Jae-in announced the New Southern Policy, marking the beginning of the Future Community Initiative implemented by Korea and the Association of Southeast Asian Nations (ASEAN). From 2018 to 2019, Korea’s official development assistance funding for Asia increased from 37 to 38.8%. In accordance with government policy, KOICA updated their regional strategy to support Vietnam, Cambodia, Laos, Myanmar, Indonesia, and the Philippines in Southeast Asia, and Uzbekistan and Mongolia in Central Asia. The number of health projects in the Asia–Pacific region has increased from 55 in 2016 to 65 in 2019 with a total budget increase from $31 million to $38 million. This trend of is expected to continue.

KOICA strives to provide quality healthcare services towards UHC in partner countries. By providing high-quality maternal, child, and newborn care services, KOICA aims to achieve SDG 3, with progress on indicator SDG 3.1.1, maternal mortality ratio, and indicator SDG 3.2.1, under-five mortality rate [16].

Kim highlighted two KOICA projects in Tanzania and Uzbekistan. Although Tanzania is not in the Asia–Pacific region, this project could be replicated. In Tanzania, KOICA is establishing comprehensive emergency obstetric and neonatal care (CEmONC) health centres. With a budget of $6.3 million, the project involves constructing CEmONC health centres, providing equipment for quality services, training health workers, and strengthening the blood transfusion system. In Uzbekistan, the Export–Import Bank of Korea has financed the construction of the Korea-Uzbekistan Friendship National Children’s Hospital. KOICA allocated $7 million to capacity building in this centre through training paediatric health professionals in advanced surgical skills.

### Japan International Cooperation Agency (JICA)

Go Tanaka, Executive Technical Advisor, Human Development Department, JICA. JICA’s global health initiative seeks to strengthen health and medical systems to achieve ‘human security 2.0’ and UHC by developing ‘hard’ infrastructures in combination with the ‘soft’ components of cooperation. Prime Minister Yoshihide outlined Japan’s priorities in leaving no one’s health behind during a 2020 UN General Assembly address. These included:

1. Developing fair and equitable access to therapeutics, vaccines, and diagnostic
2. Building hospitals by providing equipment and supporting human resource development
3. Taking action on environmental issues, such as water, sanitation, nutrition, education, and urban planning.

Tanaka outlined three pillars of action:

1. Strengthening curative health systems through constructing core hospitals, providing or rehabilitating medical equipment, and developing medical professional capacity. JICA strives to construct or renovate approximately 100 hospitals worldwide. Since 2008, JICA has provided discount financing for building hospitals throughout Asia. During this time, JICA has issued $513 million in grant aid and $774 million in loans across Asia and Oceania, with over half of the total funding directed to Southeast Asia. Responding to the need for ICU strengthening highlighted by the COVID-19 pandemic, JICA is implementing digital transformation, including telemedicine, in ICUs.
2. Enhancing research on infectious diseases and creating an outbreak alert system.
3. Promoting infectious disease prevention through education and urban planning.

JICA’s project in Mongolia provides an example of its work in improving surgical care. Funded with approximately $80 million in grant aid, the Mongolia-Japan Teaching Hospital opened in 2019. As the first university hospital in Mongolia, the 104-bed facility features advanced medical equipment and Japanese design. Surgery, gynaecology, emergency medicine, and ICU care are among the hospital’s focus areas. In addition to ‘hard’ assistance in construction and equipment, $5 million contributed to ‘soft’ support for establishing hospital management services, financial and human resource services, and patient-centred medical services.

### United States Agency for International Development (USAID)

*Monique Chireau Wubbenhorst, former Senior Advisor in Global Health, USAID* remarked that development agencies, including USAID, traditionally focus on infectious disease, maternal and child health, and nutrition. However, USAID has begun focusing on surgery through the MOMENTUM program within the past two years,. This program features a suite of maternal and child health interventions, including Caesarean sections and childbirth-associated hysterectomies for intractable postpartum haemorrhage or placenta accreta. USAID also funds surgery for blinding trachoma and lymphatic filariasis hydrocele. The COVID-19 pandemic has highlighted the need to improve critical care capacity in LMICs, including access to ventilators. This could strengthen the future of surgical care.

Private sector engagement will be the wave of the future. The size of capital markets is trillions of dollars globally, compared with only billions in the development sector. In the United States, philanthropic sector funds surpass the country’s defence budget. Many countries are scaling back development efforts; global development agency spending has plateaued over the past decade, even for infectious disease initiatives. Therefore, the private and philanthropic markets should be explored for financing opportunities. An innovative financing mechanism, revolving capital funds, could enable hospitals and facilities to acquire the capital needed to improve infrastructure. For example, USAID has worked on expanding access to oxygen treatment during the COVID-19 pandemic. The Western Pacific region has had challenges maintaining oxygen supply due to its geography and outdated infrastructure. Health facilities require capital to make needed large-scale improvements and to expand staff.

Wubbenhorst stated that while health care is often associated with the public sector, a significant proportion of care, if not the majority, is provided by the private, voluntary, and faith-based sectors. As countries become wealthier, citizens tend to seek care outside of the public sector. This trajectory can inform engagement with local organisations, especially those owning networks of hospitals and clinics, as a grassroots method of expanding surgical capacity in the developing world.

### Philanthropic organisation

#### Nick Simons Institute (NSI)

##### Anil Shrestha, Executive Director, NSI

The NSI is a family-funded, non-governmental, philanthropic organisation. It was founded by Jim and Marilyn Simons in 2006 in honour of their deceased son, Nick, whose time in Nepal fostered a love for the country and a plan to live there after completing a medical degree. The NSI is unique in that the Simons family gave the people of Nepal a ‘blank cheque’ and complete autonomy in determining the organisation’s focus. After much discussion, a group of Nepalese health professionals decided to focus on rural and curative health [17]. In these efforts, NSI supported the government health system rather than constructing independent hospitals. When the organisation was founded, only 10 to 15 of the 90 district hospitals in Nepal had functioning emergency rooms, which did not operate around the clock. Guided by Nepalese decision-makers, NSI determined to increase the capacity of district hospitals.

Initially, NSI supported three district hospitals to strengthen their health workforce. Recruiting labour for rural areas is challenging, as is the case in most LMICs. NSI offered doctors incentives and training to work in district hospitals. The organisation created a one-year anaesthesia assistant training program for general practitioners and trained them [18]. General practitioners are trained to provide anaesthesia and surgical services, including Caesarean sections, laparotomies, and simple fracture treatment. Through this, NSI worked to address the shortage of surgical, anaesthetic, and obstetric providers identified by the LCoGS [2].

Shrestha encouraged multilateral organisations to focus on surgery and curative health as well as preventive health.

### Innovative financing

#### Global Impact Partners health financing strategies

**Fig. 4:**
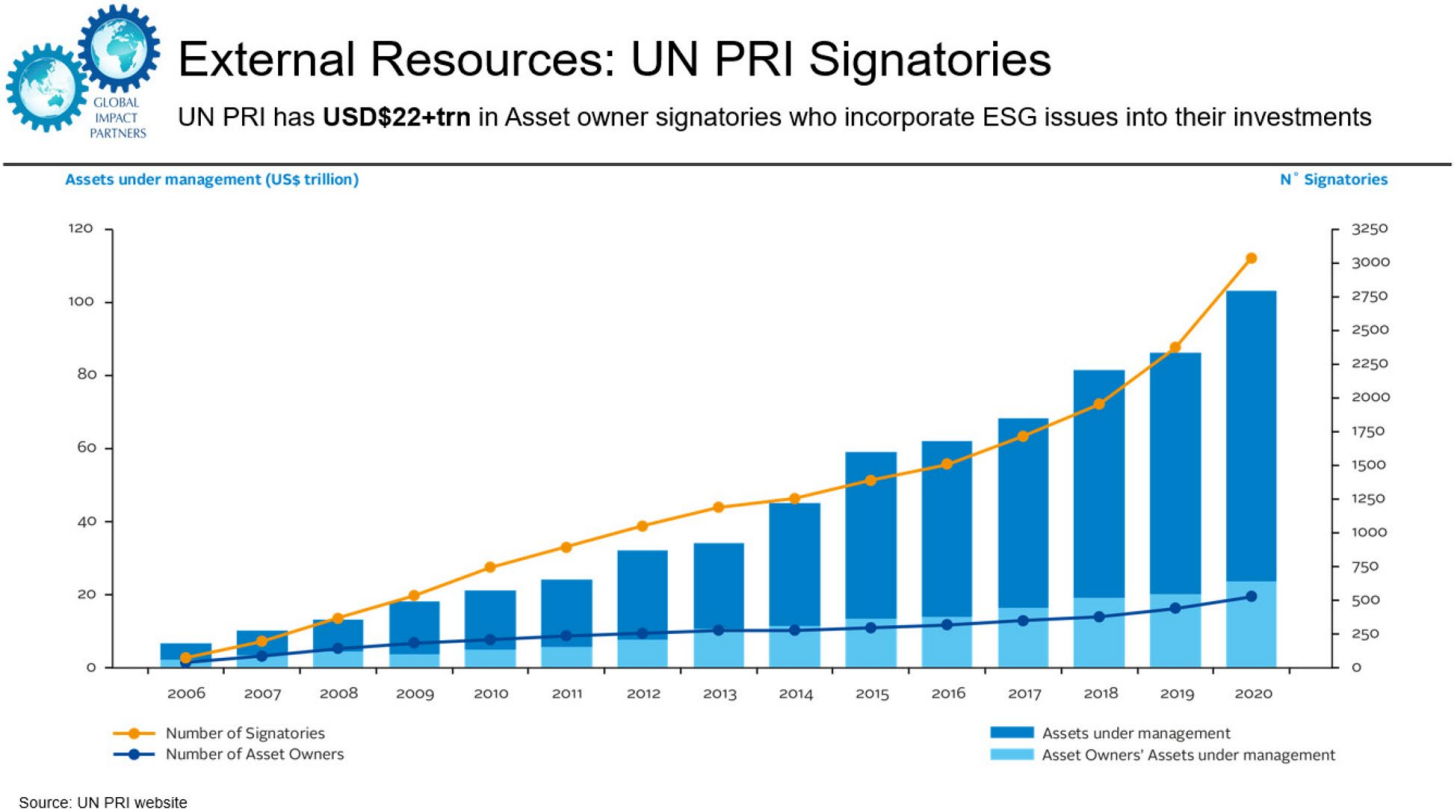
United Nations Principles for Responsible Investment signatories. UN PRI: United Nations Principles for Responsible Investment; ESG: environmental, social, and governance investing

*Shanthakumar Bannirchelvam, Managing Partner, Global Impact Partners,* stated that the mission of Global Impact Partners, an impact investment advisory firm, is to combine investment solutions with serving people and the planet. The firm operates within several sectors, including healthcare and outcomes-based financing. They collaborate with the Harvard Program in Global Surgery and Social Change (PGSSC), financial institutions, and stakeholders to identify funding gaps and opportunities to mobilise external capital to expand the fiscal space for global surgery. The asset pool for Principles for Responsible Investment signatories, which incorporate environmental, social, and governance factors into their investments, grew from zero in 2006 to over $22 trillion in 2020 (Fig. 4). Furthermore, exponential growth is taking place in investments specifically targeted for positive environmental impact. According to the Global Impact Investing Network survey, these assets totalled $710 billion in 2020 [19].

Opportunities to mobilise assets can complement efforts to advance surgery. Investible aspects of NSOAPs, including workforce development, capacity enhancement, equipment purchase, and facility investment, could be potentially funded through the private sector via institutional impact investment. The Global Health Impact partnership is designed to mobilise funds to invest in outcomes-based solutions. 134 outcomes-based financing projects have been launched in 27 countries, but few have been in health; hence, there is substantial growth potential for health projects.

An example of an outcomes-based financing health project is the Community Hypertension Prevention Initiative in Canada [20]. The Public Health Agency of Canada and the Heart and Stroke Foundation of Canada are contracted to provide hypertension prevention programs to 7,000 at-risk Canadians. An independent assessor reviewed the achievement of outcomes, and the Public Health Agency of Canada provided a return to investors. Another example involves an artificial intelligence platform, HealthPals, that analyses electronic health records to identify high-risk individuals. The United Kingdom, Australia, and India are exploring financing precision prevention efforts for cardiometabolic disease and maternal and child health. Governments can use HealthPals to identify individuals for mass prevention efforts by investing in community health worker organisations. Government health systems can then monetise downstream cost savings.

Health infrastructure financing is another key aspect in mobilising capital. The infrastructure asset class has grown exponentially over the last 30 years. Global infrastructure partners are mobilising long-term capital through infrastructure investments. The COVID-19 pandemic has demonstrated the importance of social infrastructure, which extends beyond hard infrastructure, such as toll roads, ports, and airports. Global Impact Partners is exploring opportunities with the IFC and Surbana Jurong in Singapore, India, and Indonesia. Current initiatives involve financing health clinic development by investing in the hard assets of real estate and equipment. Additionally, PGSSC is exploring using drones in India to deliver COVID-19 vaccine and blood supply to improve maternal health outcomes, particularly postpartum haemorrhage. Drone investments could remove the limitations that road infrastructure places on supply delivery. Such approaches can be considered in addressing the system blockages that limit the flow of environmentally, socially, and governmentally aligned assets into an area with shrinking fiscal space.

## Panel discussion

### Integrating surgical care at the secondary level and the growing focus on primary health care (PHC)

Samad pointed out that a large proportion of surgical care should be provided at the secondary level. She asked how this could be integrated into the shifting focus from tertiary care to PHC. Ahmed said that in order for proposals to be considered, they must be aligned with countries’ and funders’ strategies, such as the IsDB’s aim to reduce poverty. Proposals should consider and reflect economic consequences. Proposals stand a better chance of success when they include detailed implementation instructions and information regarding short-, mid-, and long-term results. IsDB also considers the income level of applicant countries. Middle- and upper-middle-income countries tend to present better proposals and secure more money. He agreed that the priority has shifted toward PHC. The PHC Performance Initiative may serve as a valuable tool at the global level.

### Aligning the interest of all stakeholders and the importance of human resource strengthening

Wubbenhorst expressed concern that funding criteria vary substantially across agencies and organisations awarding healthcare grants. For example, USAID does not fund infrastructure but funds on-the-ground training and education. She suggested that a central clearinghouse in charge of all grant monies could help connect governments and healthcare providers with the matching grantors.

To address the challenges of fragmentation, Crowley emphasised the importance of human resource strengthening, including service training, accreditation, and licensure. He also pointed out that recent investments in the health sector have all focused on COVID-19 and not on any other area of health care. When crises affect health and economics simultaneously, health often takes a lower priority than maintaining market liquidity, keeping small businesses open, and feeding citizens. He questioned how to move forward, given that the effects of COVID-19 will stretch into much of 2022; meanwhile, centres providing surgical care still need funding. *Siale Akauola, Chief Executive Officer, Ministry of Health, Tonga*, agreed with the importance of workforce strengthening. He pointed out that many Pacific Island Countries lack qualified surgeons, anaesthetists, and obstetricians who can train and mentor young specialists. Support for better mentoring and coaching is critically important.

### Strengthening surgical capacity during the COVID-19 pandemic

Samad asked how Fiji has built up capacity for bellwether procedures. Waqainabete spoke to the challenges that Ministries of Health face in dealing with normative and extra-normative functions, such as disasters and the COVID-19 pandemic. Fiji is currently running two bellwether-capable facilities, with the goal to soon run five. Fiji’s economy depends on tourism. As the COVID-19 pandemic comes to a close, Fiji must be prepared to meet tourists’ expectations in the new normal. Development partners and banks are paying attention to countries’ capacity to do so. He emphasised the importance of building health facilities in Fiji before borders fully re-open. It will ultimately support Fiji’s economic recovery.

*Benjamin Yapo, President of the Papua New Guinea (PNG) Association of Surgeons and member of the Global Initiative for Children Surgery,* explained that PNG has faced many difficulties due to the lack of funding and broken supply chain systems. The nation’s health budget had recently been cut by over $70 million, forcing at least one regional facility to close. As much as PNG may be demoralised by these events, the country is ready to improve surgical care by providing new training to surgeons and strengthening referral systems. PNG could benefit by building bellwether capability. However, the country’s geography is a significant barrier: many of the nation’s islands are isolated, and the country’s overall road infrastructure is not optimal.

### The impact of the COVID-19 response on funding for surgical system strengthening 

*Wayne Morriss, President-Elect of World Federation of Societies of Anaesthesiologists,* asked how COVID-19-related initiatives, such as increased production and supply of oxygen and ventilators, can be harnessed to support long-term surgical system strengthening. Wubbenhorst pointed out that many hospitals have rapidly increased their ventilator supply in response to COVID-19. These ventilators can be used in operating rooms. However, anaesthesia care is often a rate-limiting factor in surgical capacity. There is an opportunity for critical care training for anaesthetists to increase surgical capabilities. 

Gelb highlighted that much of the emergency provisions and funding for ventilators during the COVID-19 pandemic were temporary measures that will not be sustained in the long term. Merely teaching an individual to operate a ventilator does not make that individual an anaesthesia provider. Long-term, broad investments are required.

Alkire pointed out that few donors have the patience for meaningful long-term investments. Short- and medium-term healthcare investments are incentivised in a way that reduces the motivation for longer-term strategies. Short-term investments should be leveraged to build longer-term efforts. The mortality and GDP averted by additional ventilators and critical care nurses during the COVID-19 pandemic should be quantified. This data could be used to model the economic impact of future investments in critical care. The impacts of these short-term investments can be used as evidence to justify further investments to strengthen surgical systems after the COVID-19 pandemic subsides.

The implications of private sector funding for surgical system strengthening. 

Several participants raised concerns about private sector funding for surgical care in LMICs. A reliance on private care and inadequate insurance coverage are associated with financing barriers to care access in settings with high poverty rates. Countries should focus on providing UHC through national insurance schemes to promote equity and financial risk protection. Private investment firms are primarily motivated by generating profit for shareholders, which may not align with the priorities of recipient countries and service users. This misalignment could further disadvantage vulnerable groups.

Bannirchelvam acknowledged that not all stakeholders will align with the best interests of the global surgery community. He pointed out that cutting corners in certain areas may lead to undesirable outcomes for stakeholders with large shares in their markets. For example, under-investments in public health have ultimately led to stymied economic growth, especially during the COVID-19 pandemic. On the micro-level, incentives must be more directly aligned among funders, implementers, and recipients. Outcomes-based financing models rely on structuring contracts so that returns are delivered by improving healthcare outcomes. An accurate initial baseline is key for this approach. In Indonesia, programs are designed to decrease overall healthcare spending by preventing diabetes and reducing avoidable Caesarean sections from pre-eclampsia. He suggested transitioning the overall focus from inputs to outputs. Funds should be funnelled towards producing the best outputs rather than inputs, such as the number of nurses.

Samad replied that financing for surgical sectors is complicated. Maintaining an ongoing conversation to highlight ways to address unintended consequences is necessary.

### Making specialised and sub-specialised surgical care affordable 

Bistra Zheleva asked about strategies to finance specialised surgical procedures considered ‘unprofitable’ by hospitals and unaffordable for most people to pay out-of-pocket, such as congenital heart surgery. Kee Park, Lecturer on Global Health and Social Medicine at Harvard Medical School, said that there are pathways to include expensive items through social insurance schemes and increasing fiscal space. The Philippines was able to add transplant surgery to their national health plan using a mixture of tax and other revenues.

## Conclusion

Participants made a robust case for investing in surgical care. Three messages were derived from the discussions. Firstly, surgical, obstetric, and anaesthesia care are indispensable to UHC, cost-effective, and can yield substantial macroeconomic benefits. Secondly, whilst the COVID-19 pandemic has constricted fiscal space across the region, it has highlighted the contribution of surgical care systems to pandemic preparedness. Short-term interest in building critical care capacity must be leveraged to generate long-term investment in surgical system strengthening towards future pandemic and all-hazard emergency preparedness. Thirdly, efforts to mobilise funding for surgical care should align with funders’ priorities, which include health security, maternal and child health, primary health care, and UHC.

Whilst there are similarities between funders, there are also important differences. Participants highlighted the utility of having a mechanism to coordinate between different funders’ requirements and criteria. Whilst health funding is decreasing for some donors, it is increasing for others. Every funding source comes with strings. It is applaudable that NSI gave the people of Nepal a ‘blank cheque’ and the ability to determine their own priorities. Participants highlighted a need to build coherence between the requirements of different funders and between the goals of funders and that of recipient countries and citizens.

The session’s strength is the number and range of regional funders represented. Participants highlighted a need to examine whether shareholders’ interest aligns with service users’ interest when private capital is mobilised as additional funding sources for surgical system strengthening. This could not be fully explored given the time limit of this session and warrants further examination in future forums.

## Data Availability

N/A.

## Abbreviations

GICS: Global Initiative for Children’s Surgery
HICs: High-income countries
LCoGS: Lancet Commission on Global Surgery
LMICs: Low- and middle-income countries
NSOAP: National Surgical, Obstetric, and Anaesthesia Plan
PMA: Pasifika Medical Association
PIC: Pacific Island Country
RACS: Royal Australasian College of Surgeons
RANZCOG: Royal Australian and New Zealand College of Obstetricians and Gynaecologists
SPC: Pacific Community
UHC: Universal health coverage
UNITAR: United Nations Institute for Training and Research
WFSA: World Federation of Societies of Anaesthesiologists
WHO: World Health Organization
WHOCC: World Health Organization Collaborating Center
